# Patterns of Alcohol Consumption and Related Behaviors in Brazil: Evidence from the 2013 National Health Survey (PNS 2013)

**DOI:** 10.1371/journal.pone.0134153

**Published:** 2015-07-31

**Authors:** James Macinko, Pricila Mullachery, Diana Silver, Geronimo Jimenez, Otaliba Libanio Morais Neto

**Affiliations:** 1 University of California, Los Angeles Fielding School of Public Health, Department of Health Policy and Management, Los Angeles, CA, United States of America; 2 University of California, Los Angeles Fielding School of Public Health, Department of Community Health Sciences, Los Angeles, CA, United States of America; 3 Department of Nutrition, Food Studies, and Public Health, New York University, New York, NY, United States of America; 4 Instituto de Patologia Tropical e Saúde Pública, Universidade Federal de Goiás, Rua Delenda Rezende de Melo s/n, Setor Universitário, 74605–050, Goiania, GO, Brazil; Indian Institute of Toxicology Reserach, INDIA

## Abstract

This study uses data from a nationally representative household survey (the 2013 National Health Survey, n = 62,986) to describe patterns of alcohol consumption and related behaviors among Brazilian adults. Analyses include descriptive and multivariable Poisson regression for self-reports in the past 30 days of: drinking any alcohol, binge drinking, binge drinking 4 or more times, and driving after drinking (DD); as well as age of alcohol consumption initiation. Results show that current drinking prevalence was 26%, with an average age of initiation of 18.7 years. Binge drinking was reported by 51% of drinkers, 43% of whom reported binge drinking 4 or more times. Drinking and driving was reported by nearly one quarter of those who drive a car/motorcycle. Current drinking was more likely among males, ages 25–34, single, urban, and those with more education. Binge drinking was more likely among males, older age groups, and people who started drinking before 18. Drinking and driving was higher among males, those with more education, and rural residents. Those who binge-drink were nearly 70% more likely to report DD. All behaviors varied significantly among Brazilian states. Given their potential health consequences, the levels of injurious alcohol behaviors observed here warrant increased attention from Brazilian policymakers and civil society.

## Introduction

Alcohol consumption is associated with increased risks for a number of chronic and acute conditions as well as traffic- and violence-related injuries and deaths [1–3] While alcohol consumption is common in most of the world, there are considerable differences in the proportion and characteristics of the population that consumes alcohol, the quantity and frequency by which they consume it, and the extent to which alcohol’s potential harms may be reduced by the existence and implementation of a range of evidence-based and cost-effective programs and policies [4,5].

Brazil has the world’s fifth largest population and has experienced considerable economic growth over the past decade. As a rapidly ageing middle-income country, it faces the double burden of high rates of infectious diseases alongside climbing rates of non-communicable conditions and injuries[6]. Of the 16 countries included in a study of deaths directly attributable to alcohol, Brazil had an age-adjusted mortality rate of about 12.2/100,000, similar to that of Chile (11.6/100,000), below that observed for Mexico (17/100,000) and well above that observed in neighboring Argentina (4/100,000)[7]. Recent estimates suggest that Brazil ranks fifth in the world in traffic-related fatalities, resulting in about 40,000 deaths and 150,000 seriously injured victims annually with associated costs of nearly R$28 billion (about US$14 billion) per year [8,9]. While traffic-related mortality rates have been relatively stable since 2000, there have been recent increases, particularly in the share of deaths among motorcycle operators [10,11].

Given the high rates of traffic-related fatalities in Brazil, alcohol involvement in motor vehicle crashes bears investigating, especially in light of the country’s “dry law” (*lei seca* in Portuguese) establishing a 0.02% blood alcohol content (BAC) legal limit—equivalent to about 1 beer—for all drivers and imposing considerable penalties for BAC levels of 0.06 or above[12]. Data from the first Brazilian Household Survey of Patterns of Alcohol Use conducted in 2005/6 showed a 34.7% prevalence of self-reported drinking and driving [13]. Another study in the southern Brazilian city of Porto Alegre found that 29.6% of men and 26% of women admitted to emergency rooms for traffic accidents had consumed alcohol before the crash[14]. In contrast, very few respondents in wealthy OECD countries report having driven after consuming alcohol. While about one-fourth of all motor vehicle crash fatalities in Europe and nearly one-third each year in the US involved alcohol, the low self-report of drinking and driving may indicate how stigmatized the behavior has become in these countries [15,16].

Other patterns of alcohol use can be similarly dangerous and vary substantially among and within countries, and by socioeconomic status (SES). Behaviors such as heavy episodic (binge) drinking (drinking that brings blood alcohol concentration levels to 0.08 g/dL—usually measured as 4 drinks for women and 5 drinks for men in one setting) are strong predictors of alcohol-related mortality, morbidity, and problematic behaviors [17]. Within wealthy OECD countries individuals at higher levels of SES are more likely to consume alcohol, but to do so with greater moderation [18,19]. In contrast, those in lower socioeconomic positions in these countries may be more likely to be abstainers, but if they do drink, they tend to do so in more harmful ways. Previous work suggests this same relationship is not consistent in middle-income countries, including Brazil [20].

Demographic characteristics, such as sex and age, are also associated with drinking behaviors, and these relationships also vary among countries. In most studies, men are more likely to be drinkers and to exhibit problem drinking than women, although as countries move closer to gender equality, women begin to exhibit drinking patterns closer to those of men [21]. Younger adults are also more likely to engage in dangerous patterns of alcohol consumption and other harmful behaviors, at least in the US and some other high income countries [22]. However, in Brazil, previous studies have shown that being 30 years or younger is inversely associated with drinking and driving [13, 23, 24]. Finally, there is some evidence that drinking behaviors and their health effects may differ among racial and ethnic groups, particularly in the US, and that such differences may be related to variation in access to healthcare, differential enforcement of alcohol and impaired driving regulations, and neighborhood characteristics including the concentration of alcohol vendors within minority neighborhoods [25–28]. The relationship between race/ethnicity and alcohol has not been well explored in many other national contexts.

Given the complexity of the changing social, economic, and demographic situation in Brazil, this study explores drinking patterns using the most recent nationally representative household survey. The purpose is to describe current drinking patterns within the Brazilian population aged 18 and over, identify the prevalence and correlates of problem drinking behaviors, and to assess the relationship between these behaviors and self-reported driving after consuming alcohol.

## Materials and Methods

Data are derived from the National Health Survey (*Pesquisa Nacional de Saúde*) a nationally representative household survey conducted by the Brazilian Institute of Geography and Statistics (IBGE) and Ministry of Health in 2013. The survey employs a complex sampling design. The primary sampling units are census tracts based on the 2010 census and randomly selected from the IBGE national master sampling plan. Within each census tract households were randomly selected from a national registry of addresses. Within selected households a randomly selected respondent aged 18 or over was invited to take part in the study. The final sample size was 62,986 with an overall response rate of 78% [29]. Person-level survey weights take into account the probability of selection as well as non-response rates. The survey was approved by the Brazilian National Commission on Ethics in Research (CONEP) of the National Health Council (CNS).

We present descriptive statistics and bivariate analyses of non-drinkers versus drinkers. We then present results of multivariable Poisson regression models for each binary drinking outcome because their prevalence is well over 10% [30]. Results are in the form of incidence rate ratios (IRR). We examine 5 main outcomes: current drinkers (defined those who report having at least one alcoholic drink in the past 30 days), whether the respondent was below age 18 (the minimum legal drinking age) when they first drank, binge drinking (defined as 5 or more drinks in one setting for males and 4 for women in the past 30 days), binge drinking 4 or more times in the past 30 days, and self-reported driving after having consumed alcohol (among those who report drinking in the past 30 days and who drive a car or motorcycle). For all outcomes except current drinking, we model behaviors among current drinkers only, and for frequency of binge drinking, we model the behavior for binge drinkers only. We include age (in categories and for all models except drinking before age 18), sex, educational attainment (less than high school completed, high school completed, more than high school), self-reported skin color according to the official Brazilian census categories (white, black, pardo, Asian, Native American/Indigenous), residential location (rural versus urban and state capital residence versus elsewhere), and being married or in a relationship as control variables. To examine the relationships among drinking behaviors, we include them as covariates in subsequent models. We plot marginal effects to illustrate the effects of multiple variables on outcomes of interest.

All analyses were performed using Stata version 13.1 and results incorporate appropriate weights to control for the complex sample design. We used ArcGIS to generate maps with the prevalence of each outcome for each of Brazil’s 26 states and federal district.

## Results

Table 1 presents descriptive statistics for the sample. Slightly more than half of our sample was female, approximately one-fifth was 25–34 years of age and 12% was 65 and older. About half of respondents self-classified as white and about 40% as pardo (mixed or “brown”). Less than half had completed high school. Nearly two thirds were married or cohabiting. A quarter of the sample lived in one of the 26 state capitals or federal district, 86% resided in urban areas and nearly half of the sample reported being a driver of a car or motorcycle. More than a quarter reported having at least 1 drink in the past 30 days.

**Table 1:** Descriptive statistics of study sample.

	Total		Current non-drinker (n = 45,630)		Current Drinker^1^ (n = 14,595)	
	%	95% CI	%	95% CI	%	95% CI
Sex						
Men	47.11	[46.36,47.85]	38.95	[38.15,39.77]	69.69*	[68.29,71.05]
Women	52.89	[52.15,53.64]	61.05	[60.23,61.85]	30.31*	[28.95,31.71]
Age						
18–24	15.93	[15.36,16.51]	15.14	[14.52,15.79]	18.09*	[16.85,19.40]
25–34	21.62	[21.04,22.22]	19.86	[19.24,20.50]	26.49*	[25.23,27.79]
35–44	19.19	[18.64,19.76]	18.67	[18.00,19.37]	20.64*	[19.67,21.64]
44–54	17.50	[16.96,18.05]	17.42	[16.78,18.08]	17.70	[16.66,18.80]
55–64	13.46	[12.97,13.97]	14.24	[13.67,14.83]	11.31*	[10.44,12.25]
> 65	12.30	[11.80,12.82]	14.66	[14.04,15.30]	5.77*	[5.17,6.43]
Education						
Less than high school	38.94	[38.08,39.81]	41.76	[40.83,42.69]	31.14*	[29.73,32.59]
Less than college	43.56	[42.75,44.36]	42.57	[41.71,43.43]	46.30*	[44.71,47.89]
College or more	17.50	[16.72,18.31]	15.68	[14.92,16.46]	22.56*	[21.14,24.05]
Skin color						
White	47.46	[46.66,48.27]	46.56	[45.66,47.47]	49.95*	[48.36,51.54]
Black	9.20	[8.75,9.67]	8.95	[8.44,9.50]	9.88	[9.08,10.74]
Pardo	41.98	[41.20,42.77]	43.04	[42.16,43.92]	39.04*	[37.59,40.51]
Asian	0.94	[0.81,1.09]	1.00	[0.83,1.19]	0.78	[0.60,1.00]
Native American	0.42	[0.36,0.50]	0.45	[0.36,0.55]	0.36	[0.26,0.49]
Married/partner						
Yes	61.19	[60.45,61.92]	61.85	[61.04,62.65]	59.35*	[57.90,60.78]
No	38.81	[38.08,39.55]	38.15	[37.35,38.96]	40.65*	[39.22,42.10]
Residence						
Capital	24.72	[24.33,25.12]	24.20	[23.71,24.71]	26.16*	[25.12,27.23]
Other	75.28	[74.88,75.67]	75.80	[75.29,76.29]	73.84*	[72.77,74.88]
Region						
Urban	86.20	[85.74,86.65]	85.03	[84.42,85.62]	89.44*	[88.60,90.23]
Rural	13.80	[13.35,14.26]	14.97	[14.38,15.58]	10.56*	[9.77,11.40]
Driver	48.56	[47.71,49.41]	41.48	[40.57,42.39]	68.18*	[66.75,69.57]
Current drinker^1^	26.53	[25.70,27.28]		-		-
Age of drinking initiation, mean					18.73	[18.55,18.91]
Number of drinks consumed, mean					4.80	[4.67,4.92]
Binge-drinking, %^2^					51.47	[49.94,53.00]
Average times binge drinking/month mean^3^					3.28	[3.19,3.37]
Binge drink >4 times, %^3^					43.31	[41.29, 45.35]
Drinking and driving, %^4^					24.33	[22.67,26.06]

Results include sample weights and control for survey design.

^1^Defined as reporting having at least 1 drink in the past 30 days.

^2^At least 5 (for men) or 4 (for women) drinks in one occasion among current drinkers.

^3^ Among those reporting binge drinking in the past 30 days.

^4^ Among current drinkers who drive a car or motorcycle (n = 9,538).

*Statistically significant difference between current drinkers and non-drinkers (p<0.05).

Table 1 also shows there are substantial differences between non-drinkers and current drinkers. Compared to non-drinkers, current drinkers were predominantly male, significantly younger, more likely to self-identify as white, more likely to have started or completed college, and slightly less likely to be married or cohabiting. Current drinkers were also slightly more likely to reside in urban areas and capital cities, and were nearly 30% more likely to be drivers.

Among current drinkers, the average age of drinking initiation was 18.73 years. The average number of drinks consumed was 4.8 drinks per occasion. Binge drinking was reported by over half of current drinkers. Binge drinking occurred on average 3.3 times in the past 30 days and 43% of binger drinkers reported this behavior 4 or more times in the past month. Nearly one quarter of current drinkers who drive a car or motorcycle reported driving after drinking.

**Fig 1:**
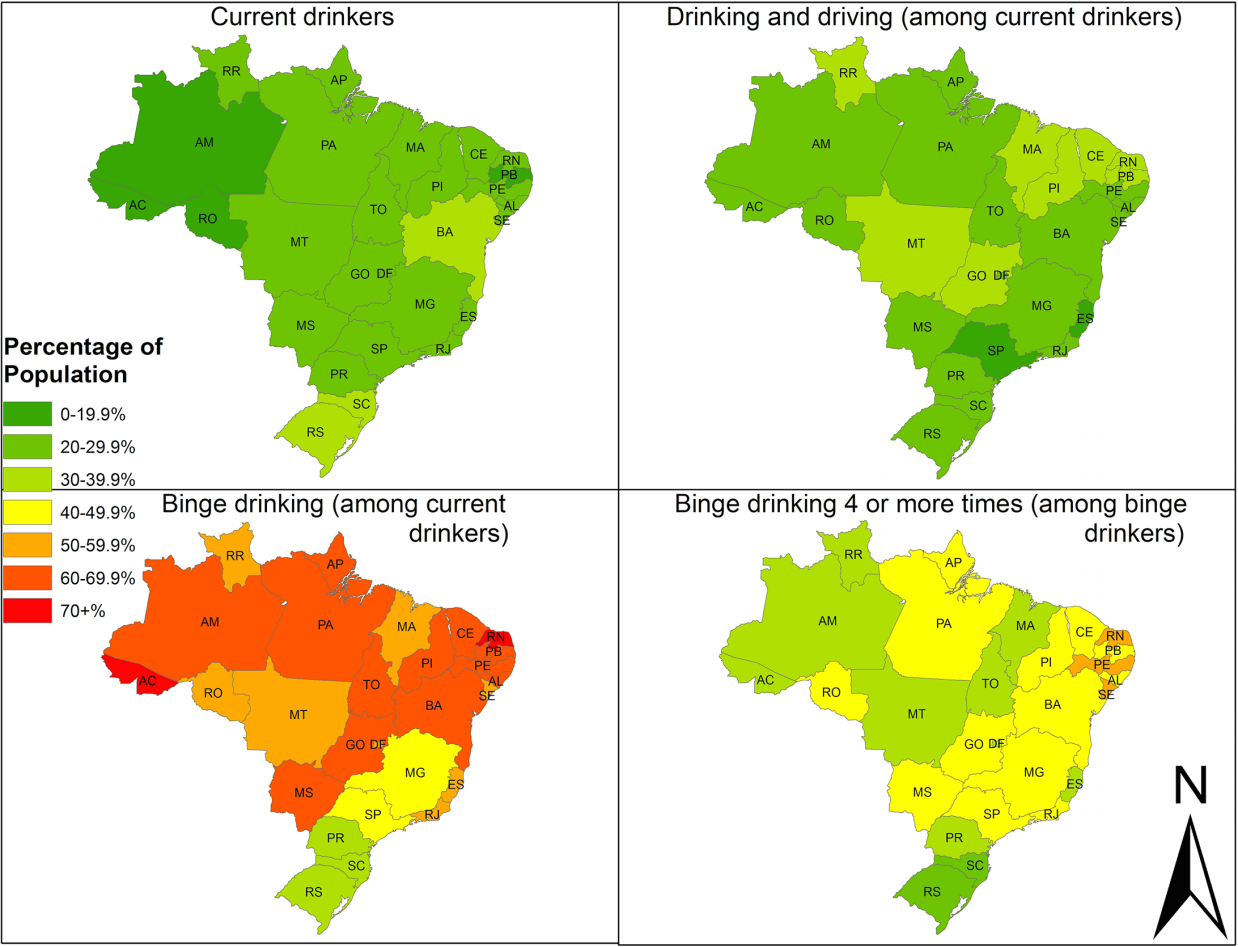
State-level prevalence rates of selected drinking behaviors among adults aged 18 and above, Brazil 2013. Data source: PNS 2013, public use Arc-GIS shape files; authors’ calculations. Results include sample weights and control for survey design. Current drinkers defined as reporting having at least 1 drink in the past 30 days. Rates for drinking and driving are among current drinkers who also drive a car or motorcycle. Binge drinking defined as at least 5 (for men) or 4 (for women) drinks in one occasion among current drinkers. Frequent binge drinking defined as 4 or more episodes in the past 30 days, among binge drinkers.

Fig 1 displays the state-level variation for four of our five outcomes within the country and highlights inconsistent patterns of drinking behaviors across states. (See S1 Table for point estimates and 95% confidence intervals for each outcome by state). The first map shows the prevalence of current drinking. The highest prevalence rates (over 30%) are seen in Rio Grande do Sul (RS), Santa Catarina (SC) and Bahia (BA), while the lowest rates are observed in the Northern region of the country. For drinking and driving (among drinkers), the map on the top right corner shows that most states had rates between 21 and 29%. The states with the highest rates of drinking and driving (between 30 and 39%) were clustered in the Northeast and Central West regions of the country. In Maranhão (MA), nearly 40% of drinkers reported drinking and driving, more than twice the rate in the prevalence states of São Paulo (SP, 18.5%) and Espirito Santo (ES, 17,1%). The bottom-left quadrant shows the percentage of drinkers who reported binge drinking in the last 30 days. Binge-drinking rates of 60% or more were observed in 14 states, mostly in the Northern and Northeastern regions. The lowest rates were observed in the Southern region of the country. The bottom right corner shows the percentage of binge drinkers who reported binge-drinking 4 or more times in the past 30 days. Sixteen of the states presented rates of 40% or more and 3 of these—all found in the Northeastern region—had rates between 50 and 59%. There was large variation among states. In Pernambuco (PE), 56.5% of the binge-drinkers reported 4 or more binging episodes, while in Rio Grande do Sul (RS), the state with the lowest rate, 22.8% of the binge-drinkers reported 4 or more episodes in the past month.

Table 2 presents results of multivariable Poisson regression models for each outcome. As seen in column 1, women were 60% less likely to be drinkers compared to men, holding constant other covariates (p<0.001). People ages 25–34 were more likely to be current drinkers than those 18–24 years, but those aged 55 and older were significantly less likely (p<0.001) to drink alcohol. The prevalence of current drinking increased with education, with college educated people being 36% more likely to be drinkers than those with less than a high school education (p<0.001). Compared to whites, people self-classified as pardo and Asian were significantly less likely to be drinkers (p<0.001). Compared to single people, those who were married or cohabitating and people living in rural areas (compared to urban) were significantly less likely to be drinkers (p<0.001). The relationship between having a drink in the past thirty days and education, race, and residence did not persist when we examined predictors of initiating drinking before age 18 (column 2). Women and those who were married or cohabitating were less likely to have initiated early drinking, all else constant (p<0.001).

**Table 2:** Predictors of drinking behaviors.

	Current drinker^1^	Drank before18	Binge drinking^2^	Binge >4 times^3^	Drove after drinking^4^
Female (v male)	0.39***	0.7***	0.79***	0.8***	0.46***
	0.37,0.41	0.65,0.75	0.75,0.85	0.71,0.89	0.38,0.56
25–34 years (v 18–24)	1.1*	-	1.13**	1.07	1.54***
	1.02,1.19		1.05,1.23	0.91,1.25	1.26,1.89
35–44	1.02	-	1.06	1.2*	1.55***
	0.93,1.11		0.96,1.16	1.02,1.40	1.26,1.91
45–54	0.96	-	0.96	1.21*	1.31*
	0.88,1.06		0.87,1.06	1.01,1.44	1.04,1.65
55–64	0.82***	-	0.69***	1.34**	1.15
	0.74,0.91		0.60,0.80	1.09,1.64	0.85,1.55
65 and older	0.48***	-	0.45***	1.25	1.05
	0.42,0.54		0.37,0.56	0.95,1.64	0.68,1.62
Less than college	1.1**	1.06	0.95	0.92	1.26**
(v < high school)	1.04,1.17	0.99,1.14	0.89,1.01	0.82,1.02	1.09,1.45
College or more	1.36***	1.07	0.84***	0.81*	1.41***
	1.27,1.46	0.98,1.17	0.77,0.91	0.69,0.95	1.18,1.69
Black (v white)	1.05	0.95	1.23***	1.1	1.05
	0.96,1.14	0.86,1.06	1.13,1.35	0.94,1.27	0.84,1.33
Pardo	0.8**	0.92	1.06	0.87	0.67
	0.64,0.99	0.69,1.23	0.81,1.38	0.55,1.39	0.37,1.23
Asian	0.89***	0.99	1.22***	1.15**	1.12
	0.84,0.94	0.93,1.06	1.15,1.30	1.04,1.27	0.98,1.27
Native American	0.82	0.83	1.13	1.42	0.59
	0.64,1.06	0.57,1.20	0.84,1.51	0.97,2.07	0.24,1.45
Married or partnered	0.91***	0.88***	0.87***	0.83***	0.85**
	0.87,0.96	0.83,0.93	0.82,0.92	0.76,0.92	0.75,0.96
Live in capital city	1.01	0.95	1.08**	1.11*	0.85*
	0.96,1.06	0.90,1.01	1.02,1.14	1.01,1.21	0.75,0.96
Rural residence	0.78***	1.04	0.92	0.88*	1.28**
	0.72,0.85	0.96,1.13	0.85,1.00	0.77,1.00	1.08,1.51
Drank <age 18	-	-	1.23***	1.16**	1.06
			1.17,1.31	1.05,1.27	0.94,1.19
Binge drinking	-	-	-	-	1.7***
					1.48,1.96
N	60,222	14,595	14,595	8,107	9,538

Results are Incidence Rate Ratios and robust 95% CIs from Poisson regression models that include sample weights and control for survey design.

* p<0.05

** p<0.01

*** p<0.001

^1^ Defined as reporting having consumed at least 1 drink in the past 30 days.

^2^ At least 5 (for men) or 4 (for women) drinks in one occasion among current drinkers.

^3^ Among those reporting binge drinking in the past 30 days.

^4^ Among current drinkers who report being car or motorcycle drivers.

Models for binge drinking show that women were about 20% less likely than men to binge-drink or to binge-drink 4 or more times in the past month (p<0.001). People aged 25 to 34 were more likely to binge-drink than younger respondents (p<0.01), while older respondents (55–64 and 64 and older) were less likely to binge-drink (p<0.001). However, among binge drinkers, the group aged 55 to 64 had the highest prevalence ratio (IRR 1.34, 95% CI 1.09,1.64) of binge-drinking 4 or more times in the previous month (p<0.01). College educated drinkers were significantly less likely to binge-drink (p<0.001) and to binge-drink 4 or more times in the past month (p<0.05), compared to those with less than a high school education. Blacks and Asians were respectively 23% and 22% more likely to binge-drink as compared to whites (p<0.001), but only Asian binge drinkers were more likely to binge-drink 4 or more times in the past month compared to whites (p<0.01). Being married or cohabitating was negatively associated with both binge drinking outcomes, as was rural residence, while living in a state capital was positively associated with both. Alcohol initiation before 18 years of age was also significantly associated with binge drinking outcomes: early age drinkers were 23% more likely on average to binge-drink (p<0.001) and these binge drinkers were 16% more likely to binge 4 or more times in the past 30 days than those who reported initiating drinking after age 18 (p<0.01).

Factors positively associated with reporting drinking and driving were somewhat different from those predicting binge drinking behaviors (last column). Among drivers, people in both the age groups 25–34 and 35–44 were over 50% more likely to drive after drinking than those aged 18 to 24 (p<0.001). The college educated were 41% more likely to report driving after drinking compared to those with a high school education (p<0.001), and rural residence was positively associated with drinking and driving (p<0.01). Factors negatively associated with self-reported drinking and driving include being female, being married or partnered, and living in a capital city. Among drivers, binge drinkers were 70% more likely to drive after drinking than non-binge drinkers (p<0.001).

**Fig 2:**
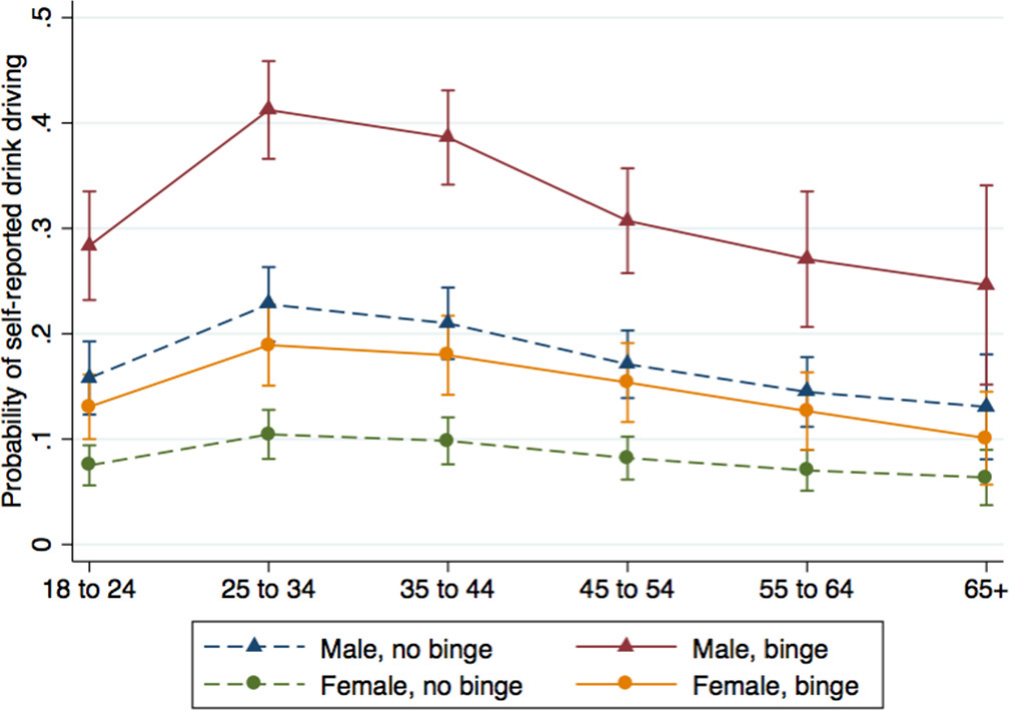
Probability of drinking and driving by report of binge drinking, sex, and age. Data source: PNS 2013, authors’ calculations. Results are predicted probabilities of self-reported drinking and driving, controlling for age, sex, educational attainment, rural and capital city residence, skin color, martial status, drinking before age 18, binge drinking, and interactions between age, sex, and binge drinking.

Fig 2 illustrates the non-linear relationships between binge drinking, age, and sex on report of drinking and driving. For both sexes, the highest adjusted rates of reporting drinking and driving occur at the ages 25–34 and 35 to 44, then decline with increasing age. Binge drinking substantially and significantly increases the probability of reporting driving after drinking among drivers-especially in the highest prevalence age group (25 to 34) where it reaches a high of nearly 40% among men. A similar pattern, albeit of lower magnitude, is seen among women.

## Discussion

Despite a prevalence of alcohol abstinence in Brazil that is significantly higher than that of the United States and most wealthy OECD countries, this study has identified high rates of injurious drinking behaviors among those who do consume alcohol [31]. More than half of current drinkers in Brazil report binge drinking and this activity occurs, on average, more than three times a month. At the high end of the distribution, nearly 20 percent of male binge drinkers and 10 percent of female binge drinkers report binge drinking 7 or more times per month. Equally alarming, nearly a quarter of current drinkers report driving after drinking.

Our findings provide a national context for city level differences between men and women observed in several studies [32–35]. Women were significantly less likely than men to report alcohol consumption at all, and among those who drank, far less likely to report drinking before age 18, binge drinking, frequent binge drinking, or driving after drinking, all else constant. Examination of interactions between education and gender (results not shown) revealed that the most educated women were almost twice as likely as the least educated to be drinkers, but were less likely to binge drink, making the drinking profile for educated women closer to that of men, albeit at much lower rates, consistent with findings in high income counties [36,37].

Those who described themselves as married or partnered were less likely to report problem drinking behaviors, a finding consistent with other international studies [31,38,39]. It may be that those able to maintain a stable relationship may be less likely to have serious drinking problems and to find support for non-problem drinking behaviors. Indeed, being married or cohabiting has been found to be indicative of alcohol abuse treatment success [39].

In contrast to age-related risk behaviors observed in other countries, the likelihood of driving after drinking was higher among adults ages 25–54, than among those ages 18–24. This difference may be the result not only of the lower likelihood of those 18–24 to be drinkers, but also reflect lesser availability of cars for younger drivers. This situation may very well change with Brazil’s rapid changing economic and social context [23]. Indeed, in other studies, an increase in purchasing power among youth has been associated with an increase in alcohol consumption. Equally noteworthy is the considerable prevalence of frequent binge drinking among those aged 35–64.

In this study, initiating drinking before age 18 was associated with a higher likelihood of binge drinking overall and binge drinking more than 4 times a month, and binge drinkers were more likely to report driving after drinking. Our findings are consistent with recent findings that among students aged 14–18 years in the 27 Brazilian state capitals, 30% of youth reported binge drinking behavior in the past year, and those with higher socioeconomic status had higher risks of binge drinking [40]. Early drinking, and particularly dangerous drinking among young people may not only increase risks of problem drinking as an adult, but has also been associated with other harmful behaviors [41].

Other studies have found racial and ethnic disparities in regards to problem drinking and this study underscored such findings. Compared to those who identified as white, Asian and mixed (pardo) individuals were less likely to be drinkers. Among drinkers, blacks and Asians were considerably more likely than whites to report binge drinking, and Asians more likely to report frequent binge drinking, even after controlling for demographic and socioeconomic characteristics. These differences merit further study since interventions to reduce problem drinking in Brazil may require adaption to different social and cultural contexts [42].

Our study provides the first picture of patterns of multiple drinking behaviors across Brazilian states and the geographic differences point to areas where alcohol control efforts should be targeted. Rural residence was associated with an increased likelihood to drive after drinking and we note high rates of self-reported drinking and driving in states with large semi-urban and rural areas. The substantive variation across states in the portion of the population that reports binge-drinking, frequent binge-drinking and drinking and driving bears further investigation. This variation may reflect differences in state populations by age distribution, levels of education, and economic factors such as car ownership. They may also suggest differences in enforcement of existing policies controlling alcohol and impaired driving.

Other studies have found self-reports to be underestimates of impaired driving [43]. Our study adds to knowledge about the perceptions of enforcement of Brazil’s “dry law” regarding drinking and driving. The finding that those living in capital cities were less likely to report driving after drinking likely reflects the fact that large cities have been more active in setting up sobriety checkpoints on the road than have municipalities in rural or semi-urban areas. Additionally, the proportion of the population who readily admit to engaging in an illegal behavior with substantial penalties suggests either a lack of awareness or concern about the law or suggests that social norms sanctioning drinking and driving have yet to fully permeate Brazilian society.

The patterns of alcohol consumption reported in this study warrant attention from Brazilian policymakers and civil society. Among drinkers, the prevalence and frequency of binge drinking and drinking after driving far exceed rates in other industrialized nations. For example, in the United States, a country with relatively high rates of binge drinking only 30% of drinkers report binge drinking. Motor vehicle crashes continue to be a leading cause of death and disability in Brazil with estimates of over 40% of traffic deaths attributed to alcohol involvement [44]. Alcohol is also strongly implicated in incidents of interpersonal violence, rates of which are disproportionately high in Brazil [6]. This clustering of dangerous alcohol-related behaviors suggests the need for continued action on Brazilian alcohol policy.

This study has some limitations. Results are based on self-report and are likely to represent underestimates of true values, especially with regard to socially sanctioned or illegal activities such as drinking and driving. However, the large sample size and state-level estimates have been able to illuminate the socio-economic, demographic, and geographic determinants of alcohol consumption and associated risk factors in a large, diverse middle income country. Because the data are cross-sectional we are unable to determine causal relationships and directionality of the observed associations. The results nevertheless point to a cluster of factors associated with elevated risky alcohol behaviors that may be targets for more detailed investigation and intervention.

## Supporting Information

S1 Table State-level prevalence and 95% CI of selected drinking behaviors, Brazil 2013. Data source: PNS 2013, authors’ calculations. Results include sample weights and control for survey design. ^1^Defined as reporting having at least 1 drink in the past 30 days. ^2^At least 5 (for men) or 4 (for women) drinks in one occasion among current drinkers. ^3^ Among those reporting binge drinking in the past 30 days. ^4^ Among current drinkers who drive a car or motorcycle.
(DOCX)
