# Supplementary material for: Patterns of Alcohol Consumption and Related Behaviors in Brazil: Evidence from the 2013 National Health Survey (PNS 2013)
Source: PLoS One. 2015 Jul 31;10(7):e0134153. doi: 10.1371/journal.pone.0134153 (PMC4521809; doi:10.1371/journal.pone.0134153)
Supplement: S1 Table — Data source: PNS 2013, authors’ calculations. Results include sample weights and control for survey design. 1Defined as reporting having at least 1 drink in the past 30 days. 2At least 5 (for men) or 4 (for women) drinks in one occasion among current drinkers. 3 Among those reporting binge drinking in the past 30 days. 4 Among current drinkers who drive a car or motorcycle. (DOCX) [file pone.0134153.s001.docx]

**S1 Table. State-level prevalence and 95% CI of selected drinking behaviors, Brazil 2013**

|  | Name | Current Drinkers^1^ | Drank before age 18 | Binge drinking^2^ | Binge >4  Times^3^ | Drinking and driving^4^ |
| --- | --- | --- | --- | --- | --- | --- |
| RO | Rondônia | 19.6 | 49.75 | 56.82 | 49.73 | 26.38 |
|  |  | [16.55,23.05] | [41.59,57.92] | [50.63,62.8] | [37.92,61.58] | [18.71,35.83] |
| AC | Acre | 17.41 | 56.67 | 71.12 | 36.90 | 25.11 |
|  |  | [15.34,19.7] | [50.4,62.74] | [63.61,77.63] | [29.06,45.49] | [18,33.86] |
| AM | Amazonas | 19.55 | 48.96 | 68.67 | 39.34 | 24.43 |
|  |  | [17.59,21.67] | [42.9,55.05] | [63.09,73.75] | [32.48,46.65] | [18.1,32.12] |
| RR | Roraima | 23.4 | 45.92 | 57.39 | 37.04 | 35.25 |
|  |  | [20.75,26.28] | [39.49,52.48] | [49.95,64.5] | [28.68,46.25] | [28.06,43.17] |
| PA | Pará | 21.43 | 48.61 | 68.82 | 45.09 | 27.88 |
|  |  | [18.7,24.43] | [42.17,55.11] | [62.22,74.73] | [36.54,53.95] | [19.53,38.1] |
| AP | Amapá | 25.32 | 50.13 | 69.45 | 42.56 | 26.37 |
|  |  | [22.11,28.83] | [42.19,58.06] | [60.68,77.01] | [31.8,54.07] | [17.27,38.05] |
| TO | Tocantins | 25.59 | 49.66 | 61.99 | 36.30 | 29.97 |
|  |  | [22.16,29.35] | [42.35,56.98] | [53.82,69.53] | [27.44,46.2] | [23.95,36.78] |
| MA | Maranhão | 23.13 | 41.85 | 56.49 | 35.21 | 38.94 |
|  |  | [20.17,26.39] | [35.66,48.3] | [48.25,64.38] | [24.56,47.57] | [30.49,48.12] |
| PI | Piauí | 26.42 | 52.83 | 64.39 | 45.58 | 37.09 |
|  |  | [23.63,29.42] | [46.96,58.61] | [58.03,70.27] | [37.92,53.45] | [30.63,44.05] |
| CE | Ceará | 21.97 | 46.5 | 64.58 | 49.86 | 30.7 |
|  |  | [19.84,24.26] | [41.07,52.02] | [58.18,70.49] | [42.45,57.27] | [23.88,38.47] |
| RN | Rio Grande | 23.38 | 52.66 | 70.39 | 50.03 | 36.07 |
|  | do Norte | [20.82,26.15] | [45.9,59.33] | [64.39,75.75] | [42.66,57.39] | [29.25,43.5] |
| PB | Paraíba | 17.43 | 53.22 | 62.69 | 43.59 | 32.89 |
|  |  | [15.11,20.03] | [46.07,60.23] | [55.94,68.98] | [33.36,54.39] | [24.56,42.46] |
| PE | Pernambuco | 24.52 | 49.77 | 61.53 | 56.46 | 22.48 |
|  |  | [22.2,27] | [44.81,54.74] | [55.85,66.9] | [49.3,63.36] | [17.3,28.69] |
| AL | Alagoas | 21.09 | 51.94 | 69.54 | 46.70 | 20.47 |
|  |  | [18.5,23.94] | [44.54,59.25] | [63.34,75.11] | [36.81,56.86] | [14.68,27.81] |
| SE | Sergipe | 26.55 | 43.13 | 56.81 | 53.21 | 24.78 |
|  |  | [23.84,29.45] | [36.24,50.3] | [51.18,62.27] | [44.78,61.47] | [18.21,32.78] |
| BA | Bahia | 30.47 | 50.31 | 61.86 | 43.82 | 27.32 |
|  |  | [27.7,33.39] | [43.67,56.94] | [56.22,67.2] | [36.4,51.51] | [18.95,37.67] |
| MG | Minas | 28.79 | 45.48 | 48.63 | 47.80 | 26.62 |
|  | Gerais | [25.14,32.74] | [41.13,49.9] | [42.77,54.53] | [39.94,55.77] | [19.82,34.75] |
| ES | Espirito | 21.83 | 44.27 | 52.59 | 30.05 | 17.11 |
|  | Santo | [18.7,25.32] | [35.17,53.78] | [44.96,60.1] | [21.1,40.84] | [11.43,24.84] |
| RJ | Rio de | 23.33 | 40.51 | 57.67 | 49.76 | 20.75 |
|  | Janeiro | [21.38,25.41] | [35.85,45.34] | [52.66,62.53] | [43.69,55.83] | [15.25,27.6] |
| SP | São Paulo | 27.12 | 46.03 | 44.76 | 43.81 | 18.51 |
|  |  | [25.37,28.94] | [42.47,49.64] | [40.76,48.83] | [38.05,49.75] | [15.27,22.25] |
| PR | Paraná | 28.61 | 44.18 | 37.02 | 33.62 | 23.48 |
|  |  | [25.84,31.54] | [38.12,50.41] | [32.01,42.32] | [24.87,43.65] | [18.05,29.95] |
| SC | Santa | 30.91 | 49.71 | 36.73 | 28.34 | 23.96 |
|  | Catarina | [26.31,35.92] | [43.14,56.29] | [30.28,43.69] | [20.69,37.48] | [18.86,29.92] |
| RS | Rio Grande | 34.55 | 53.67 | 33.01 | 22.77 | 21.77 |
|  | do Sul | [31.33,37.93] | [47.53,59.69] | [29.15,37.11] | [16.51,30.53] | [16.45,28.22] |
| MS | Mato Grosso | 29.2 | 47.41 | 62.92 | 44.89 | 26.25 |
|  | do Sul | [26.62,31.92] | [41.94,52.95] | [57.5,68.03] | [37.25,52.78] | [21.22,31.98] |
| MT | Mato | 25.36 | 36.88 | 55.24 | 35.55 | 32.86 |
|  | Grosso | [22.23,28.77] | [31.05,43.12] | [48.67,61.64] | [26.57,45.68] | [25.89,40.67] |
| GO | Goiás | 27.45 | 49.22 | 60.66 | 44.57 | 31.75 |
|  |  | [24.74,30.32] | [43.5,54.96] | [55.37,65.71] | [37.02,52.37] | [25.82,38.33] |
| DF | Distrito | 27.89 | 48.76 | 55.59 | 37.00 | 24.72 |
|  | Federal | [25.24,30.71] | [43.36,54.19] | [50.25,60.81] | [29.96,44.63] | [19.68,30.55] |
|  | Brazil | 26.53 | 47.1 | 51.47 | 43.31 | 24.33 |
|  |  | [25.79,27.28] | [45.67,48.55] | [49.94,53.00] | [41.29,45.35] | [22.67,26.06] |

Data source: PNS 2013, authors’ calculations.

Results include sample weights and control for survey design.

^1^Defined as reporting having at least 1 drink in the past 30 days.

^2^At least 5 (for men) or 4 (for women) drinks in one occasion among current drinkers.

^3^ Among those reporting binge drinking in the past 30 days.

^4^ Among current drinkers who drive a car or motorcycle.
